# Global, regional, and National Burden of chronic kidney disease attributable to dietary risks from 1990 to 2021

**DOI:** 10.3389/fnut.2025.1555159

**Published:** 2025-03-25

**Authors:** Kaixuan Wang, Shuaiqi Chen, Mengmeng Wang, Qingjiang Han, Yuchuan Hou, Xiaohui Wang

**Affiliations:** ^1^Department of Urology Surgery, The First Affiliated Hospital and College of Clinical Medicine of Henan University of Science and Technology, Luoyang, China; ^2^Department of Urology, The First Affiliated Hospital of Xinxiang Medical University, Xinxiang, China; ^3^Department of Oncology, The Second Affiliated Hospital of Henan University of Science and Technology, Luoyang, China; ^4^Department of Urology, First Hospital of Jilin University, Changchun, China

**Keywords:** CKD, disease burden, deaths, DALYs, dietary risks

## Abstract

**Background:**

Dietary risks are increasingly reported as a cause of chronic kidney disease (CKD). However, the trends in the burden of CKD attributable to dietary risks have yet to be fully elucidated.

**Methods:**

This study extracted two major indicators related to CKD caused by dietary risks from the Global Burden of Disease (GBD) database for the years 1990 to 2021, including deaths and disability-adjusted life years (DALYs). It used estimated annual percentage change (EAPC) and percentage change to assess the trends in the burden of CKD caused by dietary risks. The relationship between Socio-demographic Index (SDI) and disease burden was also further analyzed. Additionally, we utilized the contemporary age-period-cohort model from NORDPRED to project future burden of CKD attributable to dietary risks.

**Results:**

In 2021, globally, the number of deaths due to CKD caused by dietary risks was 317,010, and the number of DALYs was 7,971,281, approximately 2–3 times that of 1990, and it was expected to continue to rise before 2040. The global death rates and DALY rates of CKD related to dietary risks had increased, with EAPCs of 0.63 (95% CI: 0.57 to 0.69) and 0.39 (95% CI: 0.35 to 0.42), respectively. From a gender perspective, men were more likely to suffer from CKD due to dietary risks. From an age pattern perspective, in 2021, the number of deaths due to CKD caused by dietary risks peaked among men aged 70–74 and women aged 85–89. Additionally, the highest number of DALYs due to CKD caused by dietary risks was observed among men and women aged 65–69. In terms of socioeconomic factors, from 1990 to 2021, as the SDI increased, the age-standardized death rates and DALY rates due to CKD caused by dietary risks generally decreased. Among the seven dietary habits related to dietary risks, low vegetable intake, low fruit intake, and high sodium intake had the greatest impact.

**Conclusion:**

In summary, over the past 32 years, the burden of CKD attributable to dietary risks has rapidly increased globally, and it is expected to continue rising until 2040. Therefore, interdisciplinary actions involving education, policy, and healthcare should be taken to mitigate this growing trend.

## 1 Introduction

Chronic kidney disease (CKD) is identified by persistent abnormalities in kidney structure or function lasting over 3 months, with health implications. According to the KDIGO 2012 guidelines, diagnostic criteria include an estimated glomerular filtration rate below 60 mL/min/1.73 m^2^ and an albumin-creatinine ratio of 30 mg/g or higher (1). CKD has emerged as a critical global health issue, with its prevalence and incidence rising by 40% over the past three decades, driven by population growth and aging (2). According to the most recent Global Burden Disease (GBD) study, CKD affected 9.0% of the global population in 2019, contributing to 2.53% of total deaths and 1.64% of disability-adjusted life years (DALYs) (3). Furthermore, CKD significantly increases the risk of rapid cardiovascular disease development and progression to end-stage renal disease (ESRD). Patients with CKD or ESRD face poorer clinical outcomes, diminished quality of life, higher medical expenses, and increased economic burden (4, 5). Effective primary prevention is essential to minimize or eradicate modifiable risk factors linked to CKD.

Over the past few decades, modifiable risk factors such as diet, hypertension, impaired fasting glucose, smoking, and obesity have been identified as the primary contributors to CKD-related DALYs and mortality (2, 6). The association between dietary risks and CKD has been extensively studied, particularly in relation to high sodium diets, high protein diets, and plant-based diets. Excessive sodium consumption can lead to CKD or ESRD through various mechanisms, including elevated blood pressure, proteinuria, fluid retention, induction of inflammatory responses, oxidative stress, and endothelial dysfunction (7, 8). In addition, patients with CKD are especially vulnerable to high sodium levels because their ability to excrete sodium diminishes as kidney function deteriorates (9). For individuals with CKD or those at risk, consuming large amounts of dietary protein, particularly from animal sources, can negatively impact kidney function and long-term renal health. Recently, a study involving nearly 1800 Iranians over an average follow-up period of 6 years found that individuals consuming high-protein, low-carbohydrate diets had an increased risk of CKD (odds ratio, 1.48; 95% CI, 1.03 to 2.15) (10). Diets rich in plant-based foods may be linked to a reduced risk of CKD. Greater adherence to Mediterranean diets, which are high in fruits, cereals, vegetables, legumes, and fish, was associated with a lower incidence of CKD in a diverse cohort (11). Most importantly there is growing evidence that dietary changes may serve as effective supplementary strategies to lower the risk of negative outcomes in CKD. Medical nutrition therapy is essential for CKD patients as it can slow disease progression by carefully monitoring protein, calcium, potassium, phosphorus, and sodium levels (12). This approach alleviates symptoms without excessively restricting nutrients, thereby reducing the risk of malnutrition (13). In addition, KDOQI Clinical Guideline for Nutrition in CKD: 2020 Update recommend effective monitoring and management of the diet of CKD patients by physicians or dietitians to prevent and intervene in the progression of CKD (14).

The causes of CKD are diverse and complex, with various research approaches exploring different aspects of the disease (15, 16). To date, no studies have quantified the global burden of CKD attributable to dietary risks. Additionally, existing studies differ in their analytical methods, observation periods, and model specifications, complicating result comparisons. However, the GBD 2021 provides an opportunity to assess the impact of dietary risks on CKD burden globally, regionally, and over time, utilizing standardized methods and extensive data. Overall, this study utilized data from the GBD 2021 database to achieve the following main objectives: (1) Identify the global burden of CKD attributable to dietary risks, stratified by sex, age, GBD regions, and countries for the year 2021; (2) Analyze the trends in dietary risk-related CKD burden from 1990 to 2021; (3) Explore whether and how socioeconomic factors influence the dietary risk-related CKD burden; (4) Expand the analysis to examine the impact of seven dietary habits related to dietary risks on CKD burden; (5) Predict the trends in dietary risk-related CKD burden up to 2040. The results of this study can help policymakers identify key issues and develop public health strategies.

## 2 Materials and methods

### 2.1 Data resource and disease definition

We utilized data from the GBD 2021 database^1^ to estimate the impact of CKD attributable to dietary risks (17). This source offers the most recent epidemiological estimates on the burden of 371 diseases and injuries across 21 GBD regions and 204 countries and territories, spanning the years 1990 to 2021. All these data are freely available through the Global Health Data Exchange. The methods for data input, mortality estimation, and modeling in GBD 2021 have been extensively detailed in prior research publications, offering a thorough explanation of the techniques employed in this study (6, 18). We extracted and analyzed data including mortality, DALYs, 95% uncertainty intervals (UI), and age-standardized rates (ASR) related to CKD attributable to dietary risks from 1990 to 2021.

The definition of CKD in this research adheres to the ICD-10 (the International Statistical Classification of Diseases and Related Health Problems, 10th Revision) classification, including codes N18.1–18.5 and N18.9 (18).

### 2.2 Socio-demographic index

The Socio-demographic Index (SDI), developed by the Institute for Health Metrics and Evaluation in 2015, serves as a comprehensive measure to evaluate the development levels of countries or regions. It highlights the relationship between social progress and population health outcomes. In summary, it is the geometric mean of a 0 to 1 index, which includes the total fertility rate for individuals under 25, the average education level for those aged 15 and above, and the lag-distributed income per capita. The final range of SDI values is from 0 to 1, where 0 represents the lowest level of development and 1 represents the highest level of development. In GBD 2021, the 204 countries and territories were categorized into five SDI regions: low, low-middle, middle, high-middle, and high (17).

### 2.3 Disability-adjusted life years

DALYs, a common measure for assessing disease burden, represent the total years of healthy life lost from disease onset to death. This metric includes both Years of Life Lost (YLLs) and Years Lived with Disability (YLDs), as calculated by the following formula: *DALYs = YLLs + YLDs*. The estimates of CKD-related deaths were multiplied by the standard life expectancy estimates by age to calculate the YLLs due to CKD. The YLDs were determined by multiplying the prevalence of each CKD sequela by its respective disability weight. The total DALYs were obtained by summing the YLLs and YLDs for each CKD cause.

### 2.4 Method for forecasting CKD burden beyond 2021

Forecasting disease trends can aid in formulating effective health policies and ensuring the efficient allocation of medical resources. The R package NORDPRED utilizes the age-period-cohort model, a well-established method for predicting disease mortality and DALY. This model incorporates variables such as age, calendar period, and birth cohort to estimate death and DALY rates. The model can be succinctly represented as *R*_*αp*_=(*A*_*α*_ + *D*·p + *P*_p_ + *C*_*c*_) (5), where R_*α*p_ denotes the mortality or DALY for age group α during period p; A_α_ represents the age component for age group α; D is the common drift parameter summarizing the linear trend component; P_p_ is the non-linear period component for period p; and C_c_ is the non-linear cohort component for cohort c. Numerous studies have validated and optimized the predictive performance of NORDPRED (19). In our study, we utilized the contemporary age-period-cohort model from NORDPRED to project future burden of CKD attributable to dietary risks.

### 2.5 Statistical analysis

By accounting for standardized age structures and demographic factors, our study provides a thorough insight into the epidemiology of CKD related to dietary risks. The research utilized ASR to analyze temporal trends by determining the estimated annual percentage change (EAPC) in both age-standardized mortality rate (ASMR) and the disability-adjusted life years rate (ASDR) from 1990 to 2021. To address statistical uncertainty, all metrics were reported with 95% confidence intervals (CI). The EAPC was determined using a linear relationship model: $\ln\left(ASR\right)=\alpha +\beta x+\varepsilon$, where α is the intercept, ε is the error term, and β indicates the linear trend in ASR. The EAPC and its 95% CI were derived using the formula: $EAPC_{\text{with}95\%CI}=100\times \left(e^{\beta }-1\right)$. To evaluate trends in ASMR and ASDR, the lower boundary of the 95% CI was examined. An upward trend is indicated if this boundary is above zero, and vice versa. Additionally, Spearman’s rank order correlation was used to assess the relationship between SDI and ASMR/ASDR. Statistical significance was defined by a *p*-value <0.05. All statistical analyses were conducted using R software (version 4.4.2; https://cran.r-project.org).

## 3 Results

### 3.1 Global, regional, and National Burden of CKD attributable to dietary risks from 1990 to 2021

#### 3.1.1 Global level

**Figure 1:**
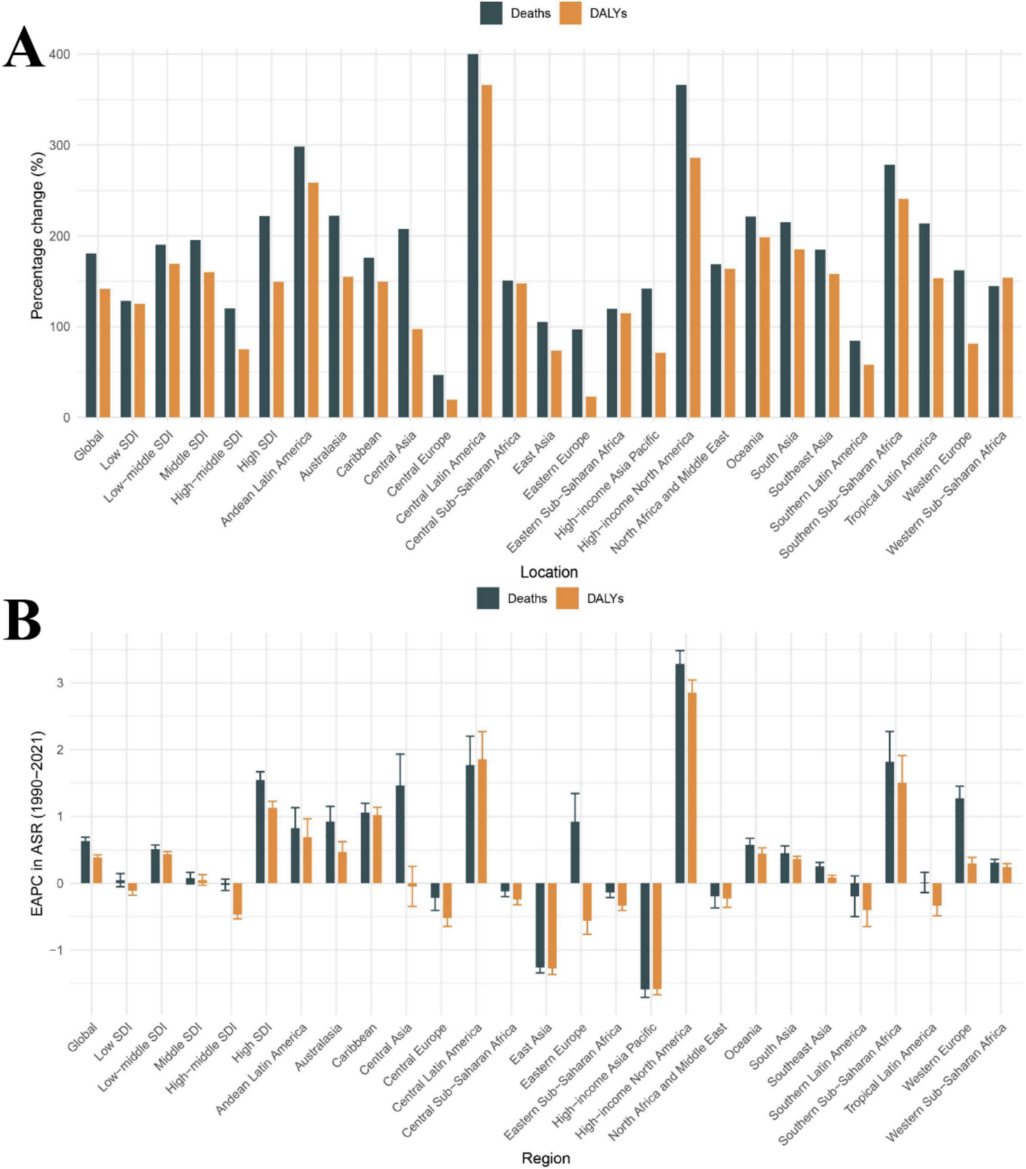
Temporal trends in CKD burden attributable to dietary risks across global, 5 SDI regions, and 21 GBD regions. **(A)** Percentage change in cases of deaths and DALYs from 1990 to 2021. **(B)** The EAPC in death rates and DALY rates from 1990 to 2021. CKD, Chronic kidney disease; DALYs, Disability-Adjusted Life Years; SDI, Socio-demographic Index; ASR, Age-standardized rate; EAPC, Estimated annual percentage change.

According to reports, the global deaths and DALYs related to CKD due to dietary risks have significantly increased. For example, the number of deaths increased from 112,949 in 1990 to 317,010 in 2021, with a percentage change of 180.67%. The number of DALYs increased from 3,299,731 in 1990 to 7,971,281 in 2021, with a percentage change of 141.57% (Table 1). Additionally, the global diet-related CKD death rates and DALY rates have both increased, with EAPCs of 0.63 (95% CI: 0.57 to 0.69) and 0.39 (95% CI: 0.35 to 0.42), respectively. This indicates that the burden of dietary risk-related CKD continues to rise globally (Table 1 and Figure 1).

**Table 1:** Global and regional indicators of CKD deaths and DALYs due to dietary risks in 1990 and 2021.

	Deaths (95% UI)						DALYs (95% UI)					
	Number (1990)	Number (2021)	Percentage change (%)	ASR/100,000 (1990)	ASR/100,000 (2021)	EAPC (1990–2021)	Number (1990)	Number (2021)	Percentage change (%)	ASR/100,000 (1990)	ASR/100,000 (2021)	EAPC (1990–2021)
Global	112,949 (66,852 to 159,069)	317,010 (185,366 to 454,851)	180.67	3.23 (1.92 to 4.57)	3.83 (2.25 to 5.49)	0.63 (0.57 to 0.69)	3,299,731 (1954,175 to 4,734,786)	7,971,281 (4,630,027 to 11,451,432)	141.57	84.12 (49.75 to 120.66)	93.52 (54.29 to 134.38)	0.39 (0.35 to 0.42)
Sex												
Male	59,107 (34,140 to 85,936)	163,980 (95,959 to 241,328)	177.43	4.00 (2.35 to 5.76)	4.59 (2.67 to 6.66)	0.52 (0.48 to 0.57)	1,768,462 (1,045,778 to 2,536,016)	4,282,936 (2,483,750 to 6,258,720)	142.18	98.95 (58.28 to 142.31)	108.58 (62.74 to 159.15)	0.37 (0.34 to 0.40)
Female	53,842 (31,691 to 77,090)	153,030 (90,263 to 221,853)	184.22	2.72 (1.63 to 3.87)	3.28 (1.94 to 4.76)	0.66 (0.59 to 0.73)	1,531,269 (880,139 to 2,167,399)	3,688,345 (2,178,868 to 5,337,947)	140.87	72.60 (41.92 to 102.87)	80.69 (47.55 to 116.70)	0.36 (0.31 to 0.40)
SDI regions												
Low SDI	12,084 (6,849 to 17,587)	27,605 (15,851 to 41,643)	128.44	6.53 (3.74 to 9.54)	6.69 (3.80 to 10.10)	0.04 (−0.06 to 0.14)	351,813 (196,418 to 510,837)	792,301 (447,547 to 1,165,725)	125.21	155.10 (88.07 to 227.53)	153.10 (87.25 to 226.01)	−0.11 (−0.18 to −0.05)
Low-middle SDI	21,111 (12,310 to 30,205)	61,307 (34,238 to 90,045)	190.40	4.04 (2.39 to 5.83)	4.78 (2.68 to 6.94)	0.51 (0.45 to 0.57)	671,791 (378,334 to 976,013)	1810,194 (1,022,368 to 2,675,561)	169.46	107.74 (61.11 to 156.87)	123.91 (69.82 to 182.13)	0.43 (0.40 to 0.47)
Middle SDI	35,984 (20,625 to 51,071)	106,368 (61,240 to 156,035)	195.60	4.33 (2.54 to 6.17)	4.37 (2.56 to 6.38)	0.08 (−0.01 to 0.16)	1,073,933 (619,491 to 1,547,179)	2,794,307 (1,599,683 to 4,027,768)	160.19	104.68 (60.14 to 151.54)	105.15 (60.22 to 151.78)	0.05 (−0.03 to 0.13)
High-middle SDI	18,703 (10,772 to 27,301)	41,193 (23,205 to 60,747)	120.25	2.19 (1.27 to 3.16)	2.15 (1.21 to 3.16)	−0.02 (−0.11 to 0.06)	565,085 (325,730 to 825,001)	987,328 (559,097 to 1,459,828)	74.72	59.10 (34.04 to 85.56)	51.19 (29.01 to 75.46)	−0.47 (−0.53 to −0.40)
High SDI	24,931 (14,841 to 35,482)	80,243 (47,008 to 114,787)	221.86	2.26 (1.35 to 3.23)	3.29 (1.92 to 4.69)	1.55 (1.42 to 1.67)	633,258 (368,324 to 906,549)	1,579,975 (951,731 to 2,229,792)	149.50	57.95 (33.65 to 83.18)	76.04 (45.79 to 106.63)	1.13 (1.03 to 1.23)
GBD regions												
Andean Latin America	1,323 (802 to 1,849)	5,269 (3,071 to 7,666)	298.26	7.18 (4.31 to 10.08)	9.27 (5.38 to 13.50)	0.83 (0.52 to 1.13)	31,467 (19,494 to 43,247)	112,838 (66,638 to 161,033)	258.59	153.17 (94.16 to 213.04)	191.06 (112.76 to 273.42)	0.69 (0.41 to 0.96)
Australasia	397 (225 to 575)	1,279 (731 to 1,809)	222.17	1.81 (1.04 to 2.62)	2.03 (1.17 to 2.88)	0.92 (0.70 to 1.15)	9,265 (5,316 to 13,380)	23,635 (13,612 to 33,508)	155.10	40.54 (23.24 to 58.83)	42.46 (24.39 to 60.99)	0.47 (0.32 to 0.62)
Caribbean	1,112 (695 to 1,539)	3,072 (1,837 to 4,413)	176.26	4.61 (2.85 to 6.37)	5.65 (3.38 to 8.11)	1.05 (0.91 to 1.20)	30,194 (18,944 to 41,263)	75,382 (46,661 to 107,582)	149.66	114.79 (71.68 to 157.67)	141.05 (87.18 to 201.24)	1.02 (0.90 to 1.14)
Central Asia	425 (212 to 678)	1,307 (652 to 2,106)	207.53	0.91 (0.46 to 1.45)	1.75 (0.87 to 2.84)	1.46 (0.99 to 1.93)	29,409 (16,192 to 44,148)	57,938 (31,241 to 89,229)	97.01	60.24 (33.09 to 91.16)	68.65 (36.41 to 106.02)	−0.05 (−0.35 to 0.25)
Central Europe	3,707 (2,024 to 5,531)	5,427 (2,872 to 8,477)	46.40	2.67 (1.46 to 3.99)	2.30 (1.22 to 3.60)	−0.22 (−0.41 to −0.03)	108,580 (59,376 to 159,583)	129,687 (70,406 to 198,941)	19.44	75.13 (41.02 to 111.06)	59.87 (32.57 to 91.84)	−0.52 (−0.65 to −0.40)
Central Latin America	4,360 (2,575 to 6,226)	21,803 (12,370 to 31,895)	400.07	6.04 (3.54 to 8.68)	8.91 (5.06 to 13.03)	1.77 (1.34 to 2.20)	122,706 (73,975 to 173,024)	572,170 (329,802 to 836,027)	366.29	144.20 (86.55 to 203.25)	224.86 (129.21 to 327.66)	1.86 (1.45 to 2.27)
Central Sub-Saharan Africa	1,728 (986 to 2,575)	4,336 (2,351 to 6,703)	150.93	10.03 (5.64 to 14.90)	10.24 (5.54 to 15.96)	−0.12 (−0.20 to −0.05)	53,083 (31,380 to 79,430)	131,454 (75,072 to 201,983)	147.64	232.44 (133.34 to 343.60)	229.23 (128.39 to 350.76)	−0.25 (−0.32 to −0.17)
East Asia	20,230 (11,417 to 30,597)	41,555 (21,338 to 64,375)	105.41	3.06 (1.75 to 4.54)	2.11 (1.08 to 3.28)	−1.26 (−1.34 to −1.18)	598,725 (335,611 to 888,395)	1,037,271 (548,156 to 1,597,294)	73.25	72.47 (41.13 to 107.44)	48.70 (25.79 to 74.68)	−1.27 (−1.37 to −1.18)
Eastern Europe	1,871 (1,055 to 2,779)	3,679 (2,031 to 5,547)	96.63	0.69 (0.39 to 1.03)	1.04 (0.57 to 1.57)	0.92 (0.50 to 1.34)	104,661 (59,856 to 151,866)	128,638 (72,469 to 189,038)	22.91	39.22 (22.20 to 57.11)	38.27 (21.62 to 56.27)	−0.57 (−0.76 to −0.37)
Eastern Sub-Saharan Africa	5,464 (3,028 to 8,201)	12,012 (6,825 to 18,474)	119.84	8.98 (5.01 to 13.59)	9.07 (5.08 to 13.90)	−0.14 (−0.22 to −0.06)	144,840 (79,246 to 216,836)	311,359 (173,379 to 472,075)	114.97	196.26 (109.00 to 296.74)	186.90 (105.39 to 283.60)	−0.34 (−0.41 to −0.27)
High-income Asia Pacific	5,960 (3,292 to 9,026)	14,419 (7,865 to 22,295)	141.93	3.36 (1.85 to 5.10)	2.16 (1.17 to 3.32)	−1.59 (−1.71 to −1.47)	143,834 (80,322 to 213,748)	245,655 (134,529 to 375,382)	70.79	74.49 (41.28 to 111.42)	46.81 (25.77 to 70.53)	−1.59 (−1.67 to −1.50)
High-income North America	8,149 (5,140 to 11,227)	38,005 (22,881 to 52,252)	366.38	2.23 (1.40 to 3.08)	5.43 (3.29 to 7.44)	3.28 (3.08 to 3.48)	202,675 (122,908 to 285,019)	782,221 (469,646 to 1,076,962)	285.95	58.04 (35.15 to 82.18)	125.15 (76.36 to 172.48)	2.85 (2.66 to 3.04)
North Africa and Middle East	5,865 (3,137 to 9,292)	15,774 (8,933 to 23,361)	168.95	4.32 (2.35 to 6.98)	4.11 (2.28 to 6.09)	−0.20 (−0.37 to −0.02)	153,304 (83,697 to 233,566)	404,333 (231,398 to 603,147)	163.75	92.87 (50.62 to 145.34)	87.79 (49.91 to 130.38)	−0.23 (−0.36 to −0.09)
Oceania	47 (24 to 75)	151 (79 to 237)	221.28	2.08 (1.05 to 3.28)	2.56 (1.37 to 3.96)	0.57 (0.48 to 0.67)	1,627 (849 to 2,543)	4,855 (2,660 to 7,618)	198.40	55.50 (29.74 to 84.75)	64.85 (35.61 to 99.93)	0.44 (0.36 to 0.53)
South Asia	15,427 (8,741 to 22,269)	48,625 (26,205 to 74,991)	215.19	3.10 (1.76 to 4.51)	3.67 (1.96 to 5.62)	0.45 (0.34 to 0.56)	553,999 (310,233 to 824,320)	1,579,713 (880,936 to 2,418,944)	185.15	92.75 (52.60 to 138.03)	105.16 (58.46 to 160.45)	0.36 (0.32 to 0.40)
Southeast Asia	13,719 (7,974 to 19,478)	39,096 (22,324 to 58,174)	184.98	6.19 (3.58 to 8.90)	6.78 (3.86 to 9.95)	0.25 (0.19 to 0.31)	401,319 (226,846 to 575,085)	1,035,742 (584,549 to 1,532,010)	158.08	152.30 (86.86 to 218.30)	157.82 (88.52 to 235.07)	0.08 (0.05 to 0.12)
Southern Latin America	2,577 (1,540 to 3,636)	4,744 (2,676 to 6,987)	84.09	6.03 (3.60 to 8.53)	5.19 (2.94 to 7.65)	−0.20 (−0.50 to 0.11)	56,578 (33,738 to 79,663)	89,278 (51,626 to 130,063)	57.80	125.07 (74.08 to 176.07)	101.68 (58.55 to 148.07)	−0.40 (−0.65 to −0.15)
Southern Sub-Saharan Africa	1,099 (601 to 1,633)	4,157 (2,317 to 5,937)	278.25	4.57 (2.51 to 6.86)	8.49 (4.72 to 12.24)	1.82 (1.36 to 2.27)	34,230 (18,971 to 51,670)	116,608 (66,285 to 167,301)	240.66	118.16 (65.44 to 177.78)	196.76 (110.55 to 283.63)	1.50 (1.10 to 1.91)
Tropical Latin America	4,097 (2,651 to 5,536)	12,855 (8,081 to 17,575)	213.77	5.13 (3.30 to 7.01)	5.16 (3.22 to 7.07)	0.01 (−0.14 to 0.16)	125,987 (79,625 to 171,460)	319,329 (199,952 to 433,078)	153.46	132.91 (83.54 to 180.64)	124.37 (77.61 to 168.66)	−0.34 (−0.49 to −0.19)
Western Europe	10,212 (6,109 to 15,002)	26,767 (15,418 to 39,855)	162.11	1.71 (1.01 to 2.51)	2.09 (1.21 to 3.11)	1.27 (1.09 to 1.45)	254,356 (145,437 to 369,054)	460,144 (264,692 to 666,728)	80.91	44.32 (25.40 to 64.81)	44.32 (25.37 to 64.23)	0.30 (0.20 to 0.39)
Western Sub-Saharan Africa	5,180 (2,863 to 7,606)	12,677 (6,902 to 19,311)	144.73	7.28 (4.11 to 10.74)	8.17 (4.44 to 12.31)	0.31 (0.26 to 0.36)	138,893 (76,832 to 202,576)	353,029 (195,123 to 528,698)	154.17	159.33 (88.06 to 233.19)	174.29 (96.00 to 261.75)	0.24 (0.18 to 0.29)

CKD, Chronic kidney disease; ASR, Age-standardized rate; DALYs, Disability-adjusted life years; EAPC, Estimated annual percentage change; UI, Uncertainty intervals; SDI, Socio-demographic index.

#### 3.1.2 SDI regional level

In 2021, the absolute number of deaths and DALYs related to dietary risk-associated CKD was highest in the middle SDI region, with 106,368 cases and 2,794,307 cases, respectively, (Table 1). Interestingly, the middle SDI region accounted for the highest proportion of global deaths and DALYs cases, approximately one-third of the total, at 33.6 and 35.1%, respectively. Additionally, the percentage change in deaths was the highest in the High SDI region, at 221.86%, while the percentage change in DALYs was the highest in the Low-middle SDI region, at 169.46% (Table 1 and Figure 1A). In contrast, the high-middle SDI region showed the smallest percentage changes, at 120.25 and 74.72%, respectively, (Table 1 and Figure 1A). Notably, from 1990 to 2021, the death rates and DALY rates in the high SDI region increased rapidly, with EAPCs of 1.55 (95% CI: 1.42 to 1.67) and 1.13 (95% CI: 1.03 to 1.23) respectively. Meanwhile, from 1990 to 2021, the high-middle SDI region showed the most significant decline in death rates and DALY rates, with EAPCs of −0.02 (95% CI: −0.11 to 0.06) and − 0.47 (95% CI: −0.53 to −0.40), respectively (Table 1 and Figure 1B). Therefore, in dietary risk-associated CKD, the middle SDI region had the largest proportion of deaths and DALYs cases, while the high SDI region experienced the most rapid increases in death and DALY rates. In contrast, the high-middle SDI region had the slowest increases in death and DALY rates.

#### 3.1.3 GBD regional level

The absolute number of deaths and DALYs related to dietary risk-associated CKD has increased over time and can be observed in all GBD regions (Table 1). Over the past 32 years, the death rates and DALY rates have continued to rise in most regions, with the largest increase observed in the High-income North America region; the EAPC for death rates was 3.28 (95% CI: 3.08 to 3.48), and the EAPC for DALY rates was 2.85 (95% CI: 2.66 to 3.04) (Table 1 and Figure 1B). In contrast, the High-income Asia Pacific region showed the most significant decline in death rates and DALY rates; the EAPC for death rates was −1.59 (95% CI: −1.71 to −1.47) and − 1.59 (95% CI: −1.67 to −1.50) (Table 1 and Figure 1B). Interestingly, the percentage change in deaths and DALYs cases in Central Latin America was particularly notable, at 400.07 and 366.29%, respectively, the highest among the 21 GBD regions (Table 1 and Figure 1A). In 2021, East Asia had the highest number of death and DALY cases, with 48,625 and 1,579,713 cases, respectively. In contrast, Oceania had the lowest number of death and DALY cases, with 151 and 4,855 cases, respectively (Table 1). In summary, 14 GBD regions have seen an increase in death rates and 11 GBD regions have seen an increase in DALY rates over time. The burden of dietary risk-related CKD in these regions is not optimistic.

#### 3.1.4 Countries level

In 2021, among 204 countries, China had the highest number of CKD deaths related to dietary risks, with 39,331 cases. In the same year, India had the highest number of CKD DALY cases related to dietary risks, with 1,226,738 cases. Notably, in 1990, China had the highest number of CKD deaths and DALY cases related to dietary risks among 204 countries, with 19,329 and 574,700 cases, respectively (Supplementary Table 1).

**Figure 2:**
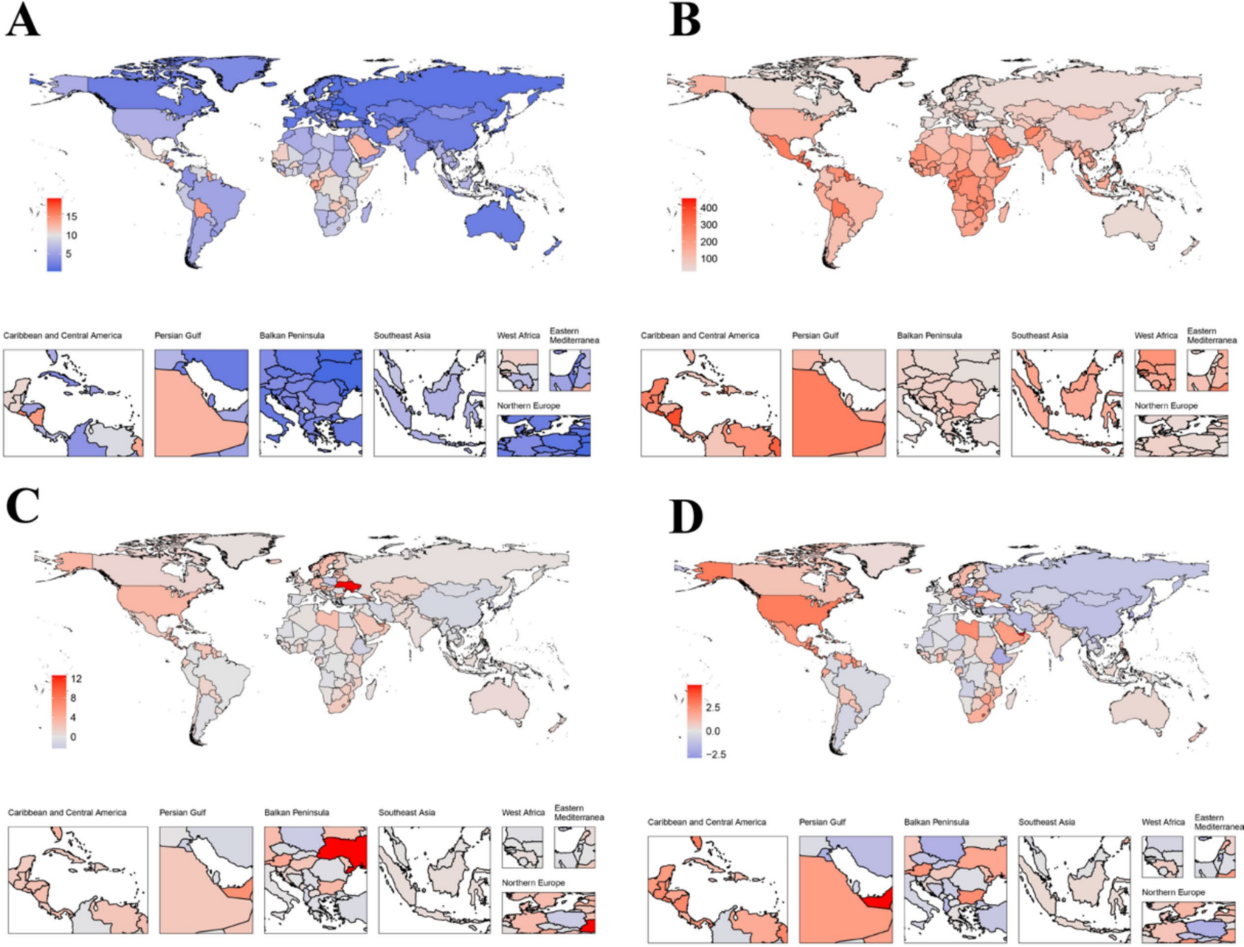
Temporal trends in CKD burden attributable to dietary risks in 204 countries. **(A)** The ASMR of CKD attributable to dietary risks across 204 countries in 2021. **(B)** The ASDR of CKD attributable to dietary risks across 204 countries in 2021. **(C)** The EAPC in ASMR across 204 countries from 1990 to 2021. **(D)** The EAPC in ASDR across 204 countries from 1990 to 2021. CKD, Chronic kidney disease; SDI, Socio-demographic index; EAPC, Estimated annual percentage change; ASMR, Age-standardized mortality rate; ASDR, Age-standardized disability-adjusted life years rates.

From 1990 to 2021, approximately 96% of countries showed an increasing trend in cases of deaths and DALYs associated with dietary risk-related CKD (Supplementary Table 1). The country with the largest percentage change in cases of deaths was Ukraine, with an increase of 1783.33%. The largest percentage change in cases of DALYs was observed in the United Arab Emirates, with an increase of 1133.43%. Interestingly, the United Arab Emirates also had a significant percentage change in cases of deaths, with an increase of 1342.86%. Additionally, Poland was the only country where the percentage change in cases of deaths and DALYs both decreased, with reductions of −10.08% and − 13.49%, respectively (Supplementary Table 1). In 1990, the countries with the highest and second highest ASMR and ASDR for CKD due to dietary risks were Ethiopia and the Maldives, respectively. Interestingly, by 2021, the countries with the highest ASMR and ASDR had changed to Mauritius and El Salvador (Figures 2A,B). Over the past 32 years, most countries have seen an upward trend in death rates and DALY rates, accounting for 75 and 63.24% of the 204 countries, respectively (Supplementary Table 1 and Figures 2C,D). For the EAPC in death rates across 204 countries from 1990 to 2021, Armenia showed the largest increase, with an EAPC of 5.7 (95% CI: 4.45 to 6.97). Additionally, for the EAPC in DALY rates across 204 countries from 1990 to 2021, American Samoa had the highest increase, with an EAPC of 3.11 (95% CI: 2.85 to 3.36). The increases in Armenia and American Samoa were significantly higher than those in high SDI regions and the North America region. Only a few countries showed a downward trend in death rates and DALY rates; the most significant decreases were observed in the Maldives, with EAPCs of −2.39 (95% CI: −2.58 to −2.19) and − 2.89 (95% CI: −3.10 to −2.67), respectively (Supplementary Table 1 and Figures 2C,D).

### 3.2 Global burden of CKD attributable to dietary risks by age and sex

We analyzed the global attributable burden of CKD caused by dietary risks by sex (Table 1 and Supplementary Figures S1, S2). From 1990 to 2021, as indicated by ASMR and ASDR, males have consistently experienced a higher burden of CKD attributable to dietary risks. Additionally, these gender differences have remained stable over the years, neither widening nor narrowing (Supplementary Figures S1, S2). On the other hand, from 1990 to 2021, the ASMR for both males and females showed a slow upward trend, with a more noticeable increase around the year 2000 (Supplementary Figure S1). No similar pattern was observed in the ASDR for males and females, with rates remaining stable from 1990 to 2021 (Supplementary Figure S2). In summary, from 1990 to 2021, both males and females have experienced a continuously increasing global attributable burden of CKD due to dietary risks, with males being more prominently affected.

**Figure 3:**
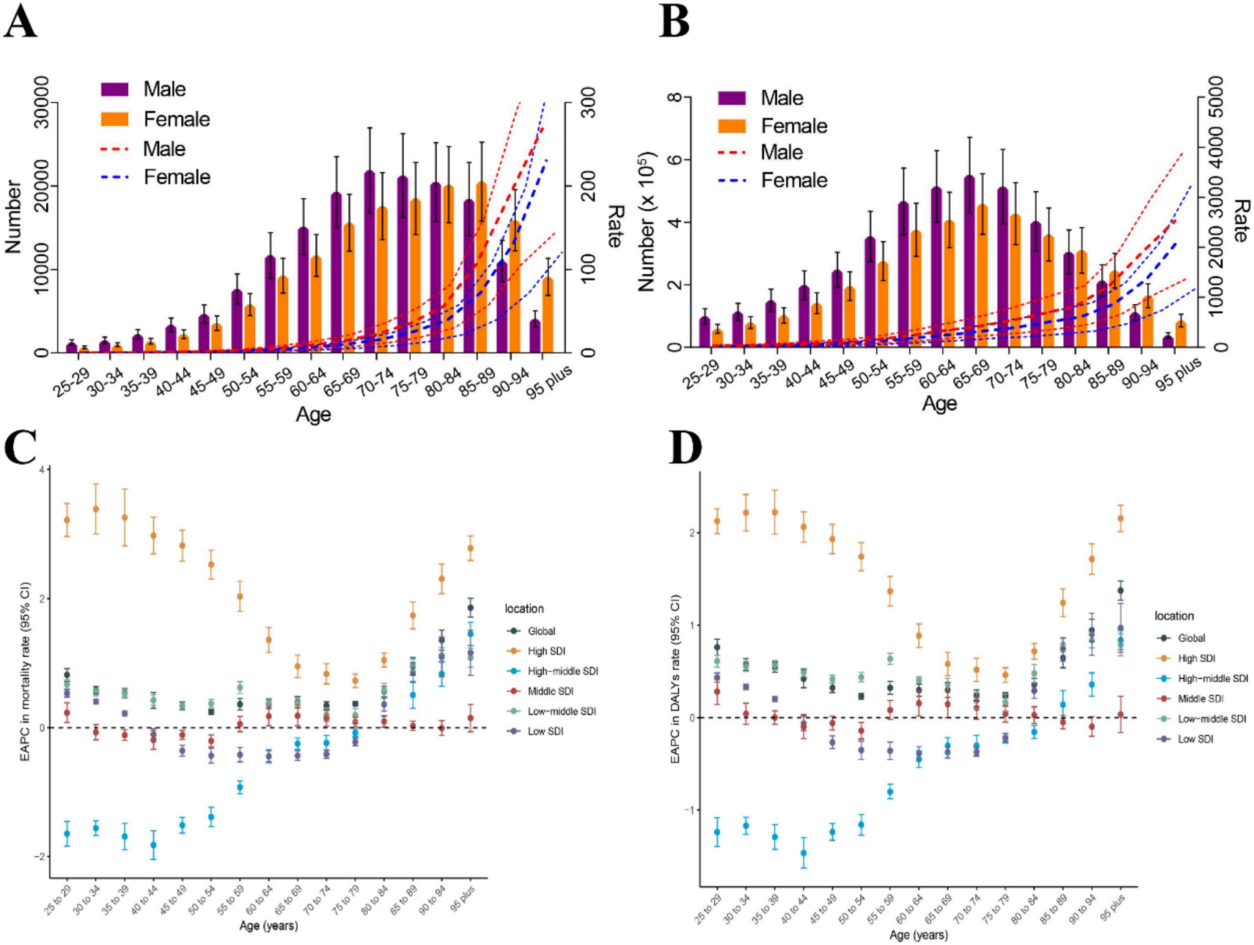
The burden of CKD attributable to dietary risks by age and sex. **(A)** Age-specific numbers and rates of CKD deaths attributable to dietary risk by sex, in 2021. **(B)** Age-specific numbers and rates of CKD DALYs attributable to dietary risk by sex, in 2021. **(C)** The age distribution of the trends in CKD-related death rate attributable to dietary risk from 1990 to 2021 by location (EAPC in death rate). **(D)** The age distribution of the trends in CKD-related DALY rates attributable to dietary risk from 1990 to 2021 by location (EAPC in DALY rates). CKD, Chronic kidney disease; DALYs, Disability-adjusted life years; SDI, Socio-demographic index; EAPC, Estimated annual percentage change.

At the same time, we also analyzed the burden of CKD attributable to dietary risks by age and sex (Supplementary Table 2 and Figure 3). In 2021, the number of CKD deaths attributable to dietary risks peaked among males aged 70–74 and females aged 85–89 (Figure 3A). The number of CKD-related DALYs attributable to dietary risks followed a normal distribution, peaking in the 65–69 age group (Figure 3B). Additionally, up to the age of 80–84, the number of CKD-related deaths and DALYs attributable to dietary risks was higher in males than in females, whereas for those aged 85 and above, the number of deaths and DALYs was higher in females (Figures 3A,B). Age-specific rates of CKD-related deaths and DALYs due to dietary risks showed a non-linear increase with age for females and males. Across all age groups, males exhibited higher CKD-related death and DALY rates attributable to dietary risks compared to females (Figures 3A,B).

In high SDI regions and low-middle SDI regions, the EAPCs in age-specific death rates and DALY rates were greater than 0 across all age groups above 25 years (Figures 3C,D). Notably, in high SDI regions, the EAPCs in age-specific death rates and DALY rates for individuals above 25 years were higher than those in the other five locations. Interestingly, in high SDI regions and low-middle SDI regions, the EAPCs in age-specific death rates and DALY rates showed an overall downward trend between the ages of 25 and 75, but the trend reverses after 75 years (Figures 3C,D). In high-middle SDI regions, the EAPCs in age-specific death rates and DALY rates increased almost linearly. From 1990 to 2021, the trend of age-specific death rate showed a downward trend among individuals aged 25–75, but an upward trend among those aged above 75. For individuals aged 25–85, the trend of age-specific DALY rates showed a downward trend, while the trend reverses for those aged above 85 (Figures 3C,D). In the middle SDI regions, the trend of age-specific death rates showed a decline in the 30–55 age group, while the trend is the opposite for other age groups. The trend of age-specific DALY rates in the middle SDI regions showed a decline in the 35–55 and 80–95 age groups, while the trend is increasing for other age groups. In the low SDI regions, the trend of age-specific death rates and DALY rates showed a decline in the 35–80 age group, while the trend is the opposite for other age groups (Figures 3C,D).

### 3.3 The association between burden of CKD attributable to dietary risks and SDI

**Figure 4:**
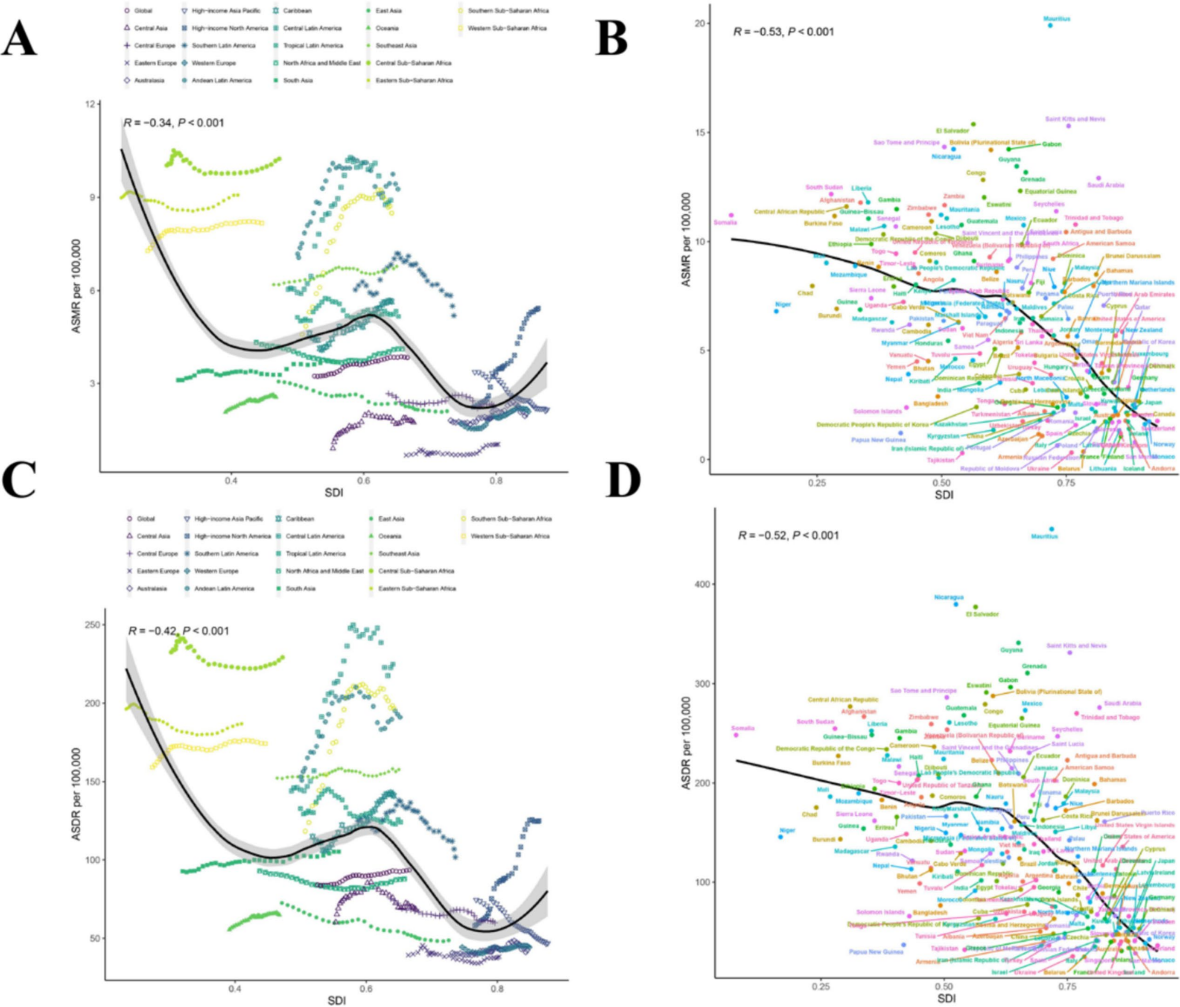
Correlations of ASMR as well as ASDR of CKD attributable to dietary risks and SDI at the regional and national level. **(A)** The ASMR of CKD attributable to dietary risks and SDI at the regional level in 21 regions from 1990 to 2021. **(B)** The ASMR of CKD attributable to dietary risks and SDI at the national level in 204 countries in 2021. **(C)** The ASDR of CKD attributable to dietary risks and SDI at the regional level in 21 regions from 1990 to 2021. **(D)** The ASDR of CKD attributable to dietary risks and SDI at the national level in 204 countries in 2021. CKD, Chronic kidney disease; SDI, The Socio-demographic index; ASDR, Age-standardized disability-adjusted life years rate; ASMR, Age-standardized mortality rate.

In 21 different GBD regions, the ASMR and ASDR of CKD attributable to dietary risks generally decreased with increasing SDI from 1990 to 2021 (Figures 4A,C; *R* = −0.34, *p* < 0.001 and *R* = −0.42, *p* < 0.001). In this context, most high-income regions were below the expected levels in all years, while many low-income regions, despite experiencing a downward trend, remained above the expected levels (Figures 4A,C). Figures 4B,D illustrated the relationship between ASMR and ASDR and SDI in different countries and regions in 2021. At the national level, ASMR and ASDR also showed an overall downward trend with increasing SDI, similar to the pattern observed in the 21 GBD regions. However, this downward trend tended to flatten before the SDI reached 0.6, and the decline became more pronounced after the SDI exceeded 0.6 (Figures 4B,D); (*R* = −0.53, *p* < 0.001 and *R* = −0.52, *p* < 0.001). This trend may be strongly linked to advancements in healthcare and economic development.

**Figure 5:**
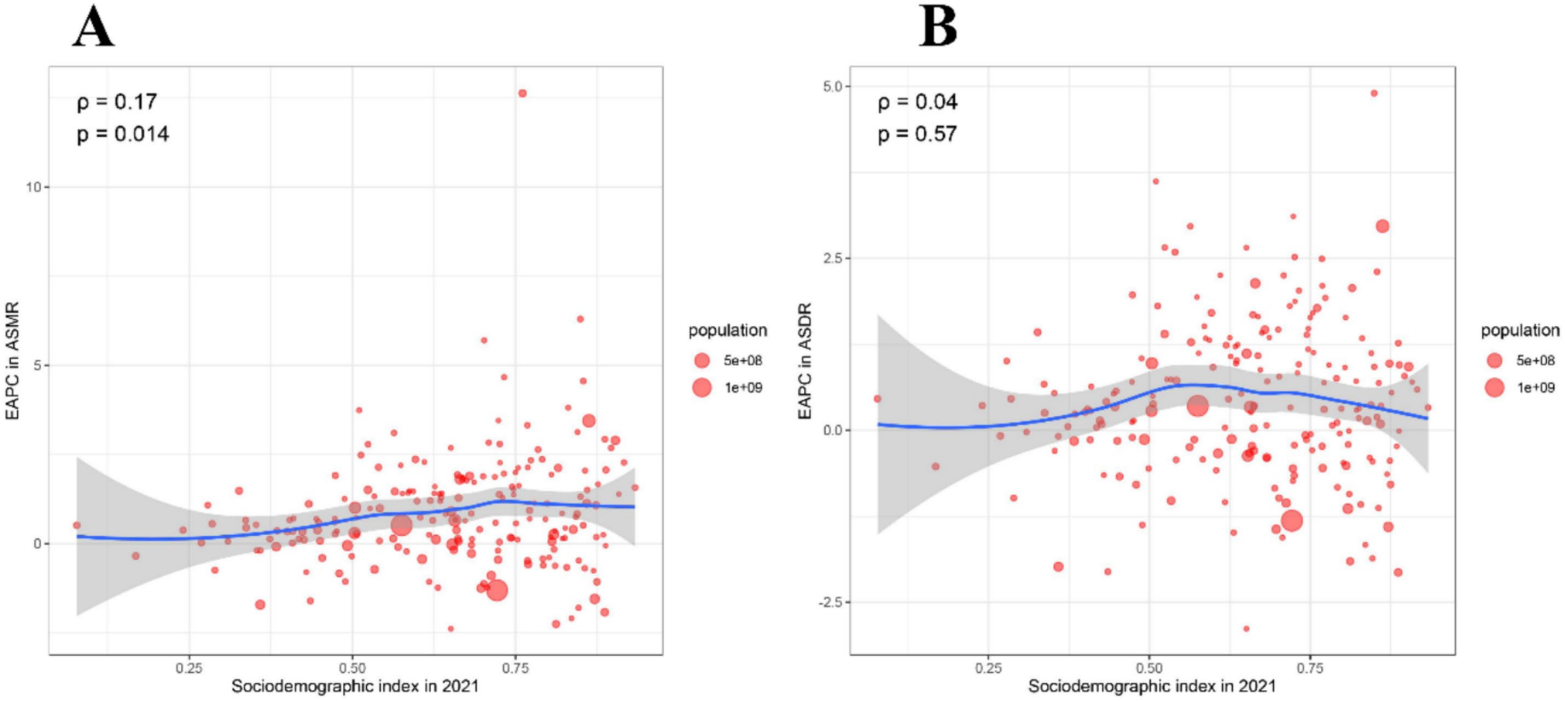
Correlation between EAPC of ASMR and EAPC of ASDR with SDI in 2021. **(A)** The correlation between EAPC in ASMR and SDI in 2021. **(B)** The correlation between EAPC in ASDR and SDI in 2021. ASMR, Age-standardized mortality rate; ASDR, Age-standardized disability-adjusted life years rate; EAPC, Estimated annual percentage change; SDI, Socio-demographic index.

We further explored the correlation between EAPC of ASMR and EAPC of ASDR with SDI in 2021 (Figures 5A,B). However, the EAPC of ASMR was slightly positively correlated with the SDI in 2021 (*ρ* = 0.17, *p* = 0.014), while the EAPC of ASDR was not correlated with the SDI in 2021 (*ρ* = 0.04, *p* = 0.57) (Figures 5A,B).

### 3.4 The association of dietary risks and seven dietary habits with CKD burden

In the global, 5 SDI regions and 21 GBD regions, we additionally explored the relationship between dietary risks and seven dietary habits with CKD-related deaths and CKD-related DALYs. Furthermore, we compared the proportion of CKD-related deaths and DALYs attributable to specific dietary components in 1990 and 2021 in global, 5 SDI, and 21 GBD regions (Supplementary Figures S3, S4). In 2021, dietary risks had a significant impact on global CKD-related deaths and DALYs, accounting for 20.8 and 17.9%, respectively (Supplementary Figures S3A, S4A). Additionally, compared to other SDI regions, the high SDI region had the highest proportion of CKD-related deaths and DALYs attributable to dietary risks. Interestingly, the proportion of CKD-related deaths and DALYs attributable to dietary risks in the high SDI region was lower in 2021 than in 1990 (Supplementary Figures S3A, S4A). In High-income North America, High-income Asia Pacific, Tropical Latin America, and Central Europe, dietary risks had a significant impact on CKD-related deaths and DALYs. Meanwhile, dietary risks had the least impact on CKD-related deaths and DALYs in the Oceania region and the North Africa and Middle East region, both in 1990 and 2021 (Supplementary Figures S3A, S4A).

The other seven dietary habits, namely diet low in whole grains, diet low in vegetables, diet low in fruits, diet high in sugar-sweetened beverages, diet high in sodium, diet high in red meat, and diet high in processed meat, also had a significant impact on global CKD-related deaths and DALYs. Among these, diet low in vegetables (6.9 and 5.9%), diet low in fruits (8.3 and 7.4%), and diet high in sodium (4.6 and 3.8%) had the greatest impact (Supplementary Figures S3B–H, S4B–H). The SDI regions exhibited different dietary risk characteristics: high-middle SDI and high SDI regions were most affected by high red meat and processed meat consumption, while high sodium intake was a major issue in middle SDI and high-middle SDI regions. In low SDI regions, insufficient consumption of fruits and vegetables constituted a significant risk (Supplementary Figures S3B–H, S4B–H). The impact of the seven dietary habits on CKD burden varied across the 21 GBD regions. Diet low in vegetables was the main risk in Andean Latin America, the Caribbean, Central Sub-Saharan Africa, Southern Sub-Saharan Africa, and Tropical Latin America. Low fruit intake primarily affected South Asia and Southern Sub-Saharan Africa. In contrast, Central Europe, East Asia, High-income Asia Pacific, and Southeast Asia faced greater risks from high sodium intake. Notably, the GBD regions most affected by diet low in whole grains and diet high in red meat were East Asia and Tropical Latin America, respectively. The GBD region most affected by diet high in sugar-sweetened beverages and diet high in processed meat was High-income North America (Supplementary Figures S3B–H, S4B–H). In summary, different SDI regions exhibit distinct dietary risk profiles, necessitating different prevention strategies. High intake of red and processed meats and high sodium intake are more pressing issues in middle SDI, high-middle SDI, and high SDI regions. Conversely, insufficient fruit and vegetable intake is a significant risk factor in low SDI regions.

### 3.5 Global disease burden prediction for CKD attributable to dietary risks

**Figure 6:**
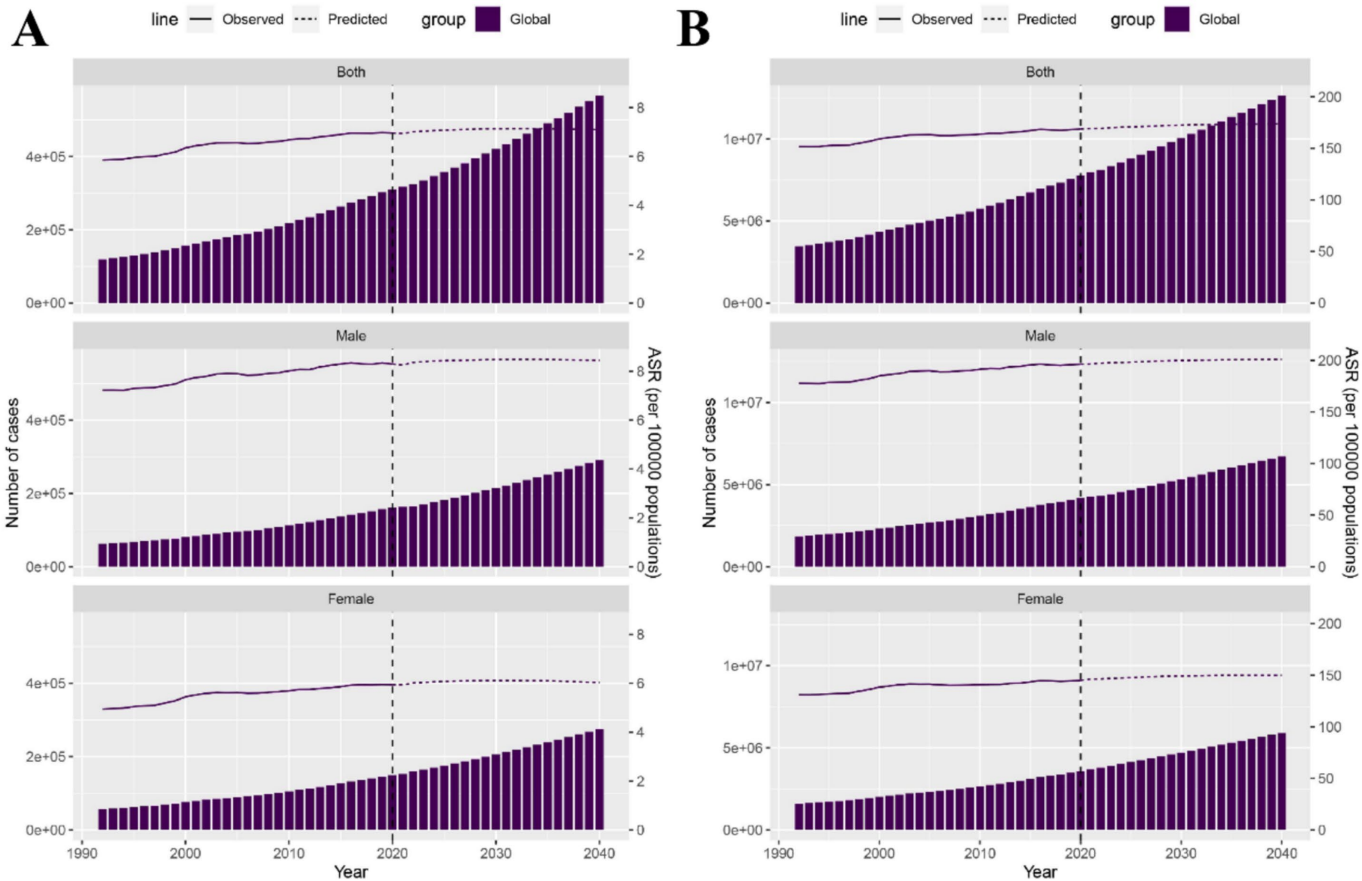
Observed and predicted trends of burden for CKD attributable to dietary risks globally from 1990 to 2040 using the NORDPRED Model. **(A)** Number of deaths and ASMR of CKD attributable to dietary risks from 1990 to 2040. **(B)** Number of DALYs and ASDR of CKD attributable to dietary risks from 1990 to 2040. CKD, Chronic kidney disease; DALYs, Disability-adjusted life years; ASDR, Age-standardized disability-adjusted life years rate; ASMR, Age-standardized mortality rate.

Based on cumulative data from 1990 to 2021, we predicted the global burden trends of CKD due to dietary risks from 2021 to 2040. According to the NORDPRED model predictions, the number of deaths and DALYs due to CKD from dietary risks will continue to increase in both male and female populations (Figures 6A,B). It is expected that during the period from 2021 to 2040, the ASMR and ASDR of CKD due to dietary risks in males will slowly rise. Meanwhile, during the same period, the ASMR and ASDR of CKD due to dietary risks in females will remain stable (Figures 6A,B). This implies that the burden of CKD due to dietary risks will continue to increase in the future, especially among the male population.

## 4 Discussion

In 2015, the United Nations introduced Sustainable Development Goal 3, which seeks to “Ensure healthy lives and promote well-being for all at all ages.” This goal aims to decrease global mortality rates and the burden of diseases by 2030 (20). Men with CKD, particularly those with dietary risk factors, are significantly aligned with this objective. CKD is prevalent and debilitating in this group, with a high incidence of associated complications, resulting in a substantial overall disease burden (21). Comprehensive understanding of the trends in CKD prevalence due to dietary risks is essential for evaluating the potential to meet related health objectives. However, there is a notable gap in comprehensive literature reviews on the deaths and DALYs associated with CKD from dietary risks across various countries and regions globally. Previous research has predominantly targeted specific age groups or individual countries (22, 23). Hence, we consider it essential to swiftly enhance and refresh the data on the burden of CKD attributable to dietary risks. This will allow policymakers to grasp the current situation and devise effective prevention and control measures. This research offers the first extensive estimate of deaths and DALYs due to CKD from dietary risks over the past 32 years globally, utilizing GBD 2021 data.

The global number of deaths and DALYs related to CKD due to dietary risks has significantly increased, changing by 180.67 and 141.57%, respectively, over the past 32 years. However, this growth may be influenced by a 45% increase in the global population. Additionally, the global deaths and DALY rates for CKD related to dietary risks have risen, indicating that the burden of CKD associated with dietary risks continues to grow. The sharp increase in CKD prevalence, impaired nutritional status due to protein-energy wasting, challenges in implementing dietary guidelines for CKD patients, and the varying dietary patterns across different regions are potential factors contributing to the ongoing increase in CKD burden (24, 25). Despite the development of diagnostic and treatment technologies over the past 32 years, the rapid increase in the burden of CKD related to dietary risks remains unresolved. When we extend the burden of CKD related to dietary risks to 5 SDI regions, 21 GBD regions, and 204 countries, we obtain key information: the death and DALYs ratios grow fastest in high SDI regions, high-income North America, and Armenia, and the proportion of death and DALYs cases is largest in middle SDI regions. Continuous monitoring of trends in these regions and countries is urgently needed, and these regions and countries should actively learn from the prevention and control strategies of better-performing regions. Additionally, some regions (such as High-income Asia-Pacific) have declining CKD mortality rates, while others (such as High-income North America) have rising mortality rates. Although these regions are both high-income, they exhibit opposite trends. We consider that different cultural dietary habits and healthcare policies may play important roles. The High-income Asia-Pacific region places more emphasis on low-protein diets (e.g., recommended protein intake for CKD patients is 0.6–1.2 g/kg/day) and uses starchy foods to replace traditional staples, reducing the intake of non-quality protein, which may help delay kidney function deterioration. In contrast, the high-protein diet culture in High-income North America (e.g., red meat, processed foods) may increase the burden on the kidneys, and the high obesity rate will further exacerbate CKD progression. Additionally, High-income North America may face disparities in healthcare resource allocation or insufficient healthcare coverage for minority groups, leading to high-risk CKD populations (e.g., patients with diabetes and hypertension) not being promptly intervened, accelerating CKD progression (26, 27).

In previous studies, researchers have often found that women are more susceptible to kidney impairment. In global, 336 million men and 417 million women have impaired kidney function, yielding a ratio of 0.81. The study also reports that 1.7 million men and 1.3 million women are treated with dialysis, resulting in a ratio of 1.3. Additionally, 0.4 million men and 0.3 million women have a functioning kidney transplant, with the same ratio of 1.3 (28). CKD stages 3–5 are more prevalent in women, while men exhibit a higher prevalence of albuminuria and CKD stages 1–2. Men experience a more rapid decline in renal function, progress to kidney failure more often, and have greater mortality and cardiovascular risk compared to women (29, 30). In our study, in 2021, the number of deaths from CKD caused by dietary risks peaked among men aged 70–74 and women aged 85–89, further demonstrating that men experience faster declines in kidney function, more frequent progression to kidney failure, and higher mortality. Therefore, early intervention (especially during youth and middle age) can theoretically reduce the future burden of CKD. Long-term intake of excessive salt (e.g., processed foods), excessive protein (especially animal protein), or excessive sugar from youth to middle age can lead to the cumulative effects of long-term metabolic load. This cumulative effect of metabolic load further increases the risk of hypertension, obesity, and glomerular filtration load, ultimately accelerating kidney function decline (31–33). Therefore, during childhood and adolescence, school nutrition programs should limit sugary drinks and processed foods, promote low-salt diets, and minimize early occurrences of obesity and hypertension to reduce long-term CKD risk. In middle age, individualized nutritional management (e.g., replacing animal protein with plant protein, limiting sodium intake to <2 g/day) for high-risk groups (such as those with hypertension and prediabetes) is needed to slow the rate of kidney function decline. At the same time, we propose an interesting point that the burden of CKD caused by dietary risks is more likely to occur in the male population rather than the female population. This is largely related to men’s worse eating habits, unhealthy dietary patterns, and unbalanced nutrition, which is reflected in the higher levels of albuminuria found in men (34). Men generally consume more red meat, processed foods, and high-sodium foods (such as fast food and pickled foods), while women prefer vegetables, fruits, and low-fat diets. Women are more likely to seek medical help earlier due to reproductive health management and attention to chronic symptoms (like edema and fatigue), leading to a higher CKD diagnosis rate. Men often delay seeking medical attention, resulting in more advanced CKD stages at diagnosis. Additionally, Estrogen has protective effects in animal experiments, suggesting it can delay kidney fibrosis by inhibiting the renin-angiotensin system (RAS) activity and reducing oxidative stress (34, 35). Therefore, targeted gender-specific interventions are crucial: (1) Promote low-sodium meals in male-dominated industries (e.g., construction, transportation), (2) Spread “less salt, less alcohol” messages via sports events and social media, (3) Enhance blood pressure monitoring and alkaline diet guidance for women over 45 (e.g., increase citrus fruit intake), (4) Integrate CKD screening into maternal and child health programs.

As CKD is an age-related disease, we have conducted a global burden analysis focusing on the different age groups and the risk of CKD caused by dietary risks. The prevalence and death of CKD is significantly higher in the elderly population, with 33.24% of individuals aged 65 and older affected, compared to only 9.04% of adults under 65, according to the USRDS (36). Previous studies have been limited in scope, but we are the first to quantify the global burden of CKD due to dietary risks. As the global population continues to age, the number of individuals aged 65 and older is projected to rise from 703 million in 2019 to over 1.5 billion by 2050, leading to an anticipated increase in the burden of CKD (37). We hope this study will serve as a wake-up call for more researchers to pay attention to the increasing burden of CKD among the elderly. Additionally, we predicted that by 2040, the CKD burden due to dietary risks would continue to rise. The NORDPRED model we used was based on the APC framework, which aimed to predict disease burden by decomposing age, period, and cohort effects. Decomposing these effects helped to distinguish changes in age structure from other risk factors. Population aging was explicitly incorporated through the model’s age effect and future population structure data, serving as a structural driver of disease burden growth. Dietary risk factors, on the other hand, were key modifiable drivers of the increase in burden. Therefore, both population aging and dietary risk factors played significant and combined roles in the prediction results. These made our prediction results more accurate and convincing. Improved detection levels might have caused short-term fluctuations in the burden, but this effect was minimal in long-term predictions.

Generally, higher SDI levels are associated with higher quality medical services and stronger healthcare systems, thereby reducing the disease burden. Across 21 different GBD regions and 204 countries, from 1990 to 2021, the ASMR and ASDR of CKD due to dietary risks generally decreased with increasing SDI. The most reasonable hypothesis for this result is that higher SDI levels are often accompanied by the dissemination of medical knowledge, allowing CKD related to dietary risks to be diagnosed and treated early. Conversely, people with lower SDI levels find it more difficult to access a diet that meets the body’s nutritional balance. They tend to favor energy-dense diets, which inevitably bring corresponding dietary risks. Additionally, influenced by local cultural backgrounds, some populations are more inclined towards certain types of food, neglecting the interactions between various nutrients (38, 39).

The 2020 KDOQI guidelines recommend the Mediterranean diet for adults with CKD stages 1–5 who are not on dialysis, as well as for transplant patients, to enhance lipid management. For CKD stages 1–4, increasing the intake of fruits and vegetables is advised to help lower body weight, blood pressure, and acid load production. Similarly, an alkaline diet is suggested for CKD stages 1–4 to slow the decline in GFR (14). Fruits and vegetables provide essential dietary fiber. Consuming about 27 g/day of fiber can reduce serum urea and creatinine levels in CKD patients, as high levels of these markers indicate abnormal GFR (40, 41). A diet rich in vegetables and fruits, high in fiber, and low in protein can positively alter the gut microbiome, modulate uremic toxin production, and slow CKD progression while reducing cardiovascular risk (22, 42). High dietary sodium is a significant factor affecting blood pressure, leading to salt-sensitive hypertension and fluid retention in CKD patients (43). It also directly contributes to renal damage. Animal studies have shown that high salt intake invariably worsens kidney damage, with salt-restricted diets linked to reduced glomerulosclerosis and proteinuria in unnephrectomized spontaneously hypertensive rats (44). High sodium intake causes hyperfiltration, which can result in renal damage, as demonstrated in numerous studies (45). Additionally, a recent double-blind, randomized controlled trial in CKD patients (stages 3 and 4) revealed that dietary sodium restriction significantly lowered ambulatory BP by 10/4 mmHg, along with consistent reductions in proteinuria and albuminuria (46). In our study, low vegetable intake, low fruit intake, and high sodium intake were identified as the three most significant factors affecting global CKD-related deaths and DALYs, which is highly consistent with the aforementioned research. More importantly, to date, our study is the only one that has quantified the global burden of chronic kidney disease attributable to dietary risks. Although the risk of a high-sugar diet does not contribute significantly to the overall CKD disease burden, high sugar intake has been identified as a risk factor associated with CKD-related conditions such as obesity, hypertension, and diabetes (47). Furthermore, several clinical studies have shown a link between sugar consumption and kidney damage (48, 49). The results of this study show that, with the exception of Central Sub-Saharan Africa, the proportion of CKD attributed to high sugar intake increased in all regions in 2021 compared to 1990. Therefore, the impact of sugar on the kidneys should not be ignored, and limiting sugar intake is crucial for maintaining health. High-protein diets, particularly those rich in meat, not only increase the risk of cardiovascular disease but also elevate CKD incidence and accelerate its progression as a result of increased intraglomerular pressure as well as glomerular hyperfiltration. Meat consumption raises the production of nitrogenous waste, exacerbates uremia, and may lead to constipation, resulting in hyperkalemia due to typically low fiber intake (50, 51). Additionally, we should focus on the increasing burden of CKD caused by a diet high in red meat and a diet high in processed meat in countries and regions with high SDI levels. High SDI regions should focus on reducing the intake of red meat and sodium. Low SDI regions should prioritize improving the accessibility of fruits and vegetables and supplementing with low-cost alkaline foods (such as legumes). Additionally, there is significant room for improvement in the dietary patterns of different countries. For countries like the United States and Germany, which have excessive consumption of red meat/processed meat and high-sugar beverages, it is recommended to enforce warnings on the packaging of products such as bacon and sausages, stating “excessive consumption increases the risk of CKD,” and to implement tiered taxes on sugary beverages to subsidize healthy foods for low-income groups. For countries like Japan and China, where high sodium intake is caused by excessive use of soy sauce and takeout food, it is advised to develop low-sodium soy sauce and promote low-salt meals through media campaigns. For regions with economic constraints, such as South Asia and Southern Sub-Saharan Africa, it is necessary to improve the supply of fruits and vegetables through agricultural subsidies and community garden projects and incorporate these foods into school meal programs. Future improvements in interdisciplinary collaboration between urologists and dietitians may improve the burden of CKD due to dietary risks. Ultimately, it is crucial to create targeted prevention and treatment strategies tailored to the specific burden of this disease.

## 5 Limitation

This study has several limitations. Firstly, the data utilized originates from GBD 2021, an online resource that offers projections on disease burden rather than real-time data from monitoring and surveillance. Secondly, the causes of CKD are multifaceted, and although GBD 2021 excluded other risk factors when analyzing dietary risk exposure, attributing the development of this chronic condition to a single factor is often inadequate. Furthermore, due to the inherent limitations of the multiple methods used in this study, it is not possible to accurately present more results. Lastly, due to differences in disease registration policies among countries, high-quality primary data are rarely available in underdeveloped countries. As a result, the GBD study relies on statistical methods and predictive covariates to generate final estimates. Consequently, variations in data quality, accuracy, and comparability may lead to biases in the final estimates.

## 6 Conclusion

In summary, over the past 32 years, the burden of CKD attributable to dietary risks has rapidly increased globally, and it is expected to continue rising until 2040. From a gender perspective, males are more susceptible to CKD burden due to dietary risks. In terms of age patterns, in 2021, the number of deaths from CKD caused by dietary risks peaked among males aged 70–74 and females aged 85–89. Additionally, the DALYs attributable to dietary risk-induced CKD reached their highest among both genders aged 65–69. Regarding socioeconomic factors, from 1990 to 2021, as the SDI increased, the age-standardized mortality rate and DALY rates of CKD due to dietary risks generally declined. Among the seven dietary habits related to dietary risks, low vegetable intake, low fruit intake, and high sodium intake had the most significant impact. Therefore, interdisciplinary actions involving education, policy, and healthcare should be taken to mitigate this growing trend.

## Data Availability

The original contributions presented in the study are included in the article/Supplementary material, further inquiries can be directed to the corresponding authors.

## Footnotes

^1^ https://ghdx.healthdata.org/gbd-2021/sources
