# Supplementary material for: Global, regional, and National Burden of chronic kidney disease attributable to dietary risks from 1990 to 2021
Source: Front Nutr. 2025 Mar 25;12:1555159. doi: 10.3389/fnut.2025.1555159 (PMC11975581; doi:10.3389/fnut.2025.1555159)
Supplement: Supplementary file 2 [file Image_1.pdf]

Supplementary Figures

Contents

Supplementary Figure S1. Global attributable burden of CKD attributable to dietary risks by sex. (the age-standard ASMR)

Supplementary Figure S2. Global attributable burden of CKD attributable to dietary risks by sex. (the age-standard ASDR)

Supplementary Figure S3. Proportion of CKD-related Deaths attributable to specific dietary components in 1990 and 2021,

Supplementary Figure S4. Proportion of CKD-related DALYs attributable to specific dietary components in 1990 and 2021,

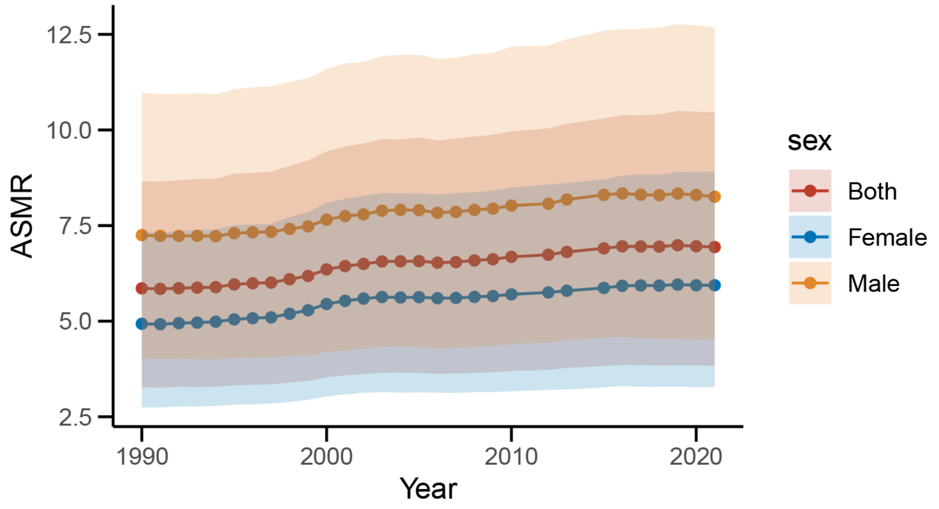

Supplementary Figure S1. Global attributable burden of CKD attributable to dietary risks by sex. The age-standard ASMR of CKD attributable to dietary risks by sex from 1990 to 2021. ASMR, age standardized mortality rate; DALYs, disease adjusted life year. CKD, Chronic kidney disease;

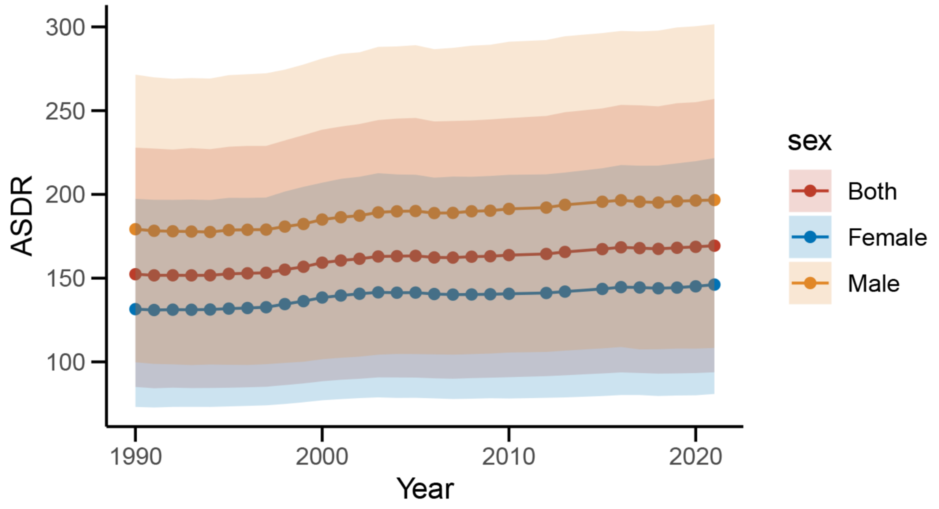

Supplementary Figure S2. Global attributable burden of CKD attributable to dietary risks by sex. The age-standard ASDR of CKD attributable to dietary risks by sex from 1990 to 2021. CKD, Chronic kidney disease; DALYs, disease adjusted life year. ASDR, age standardized DALYs rate.

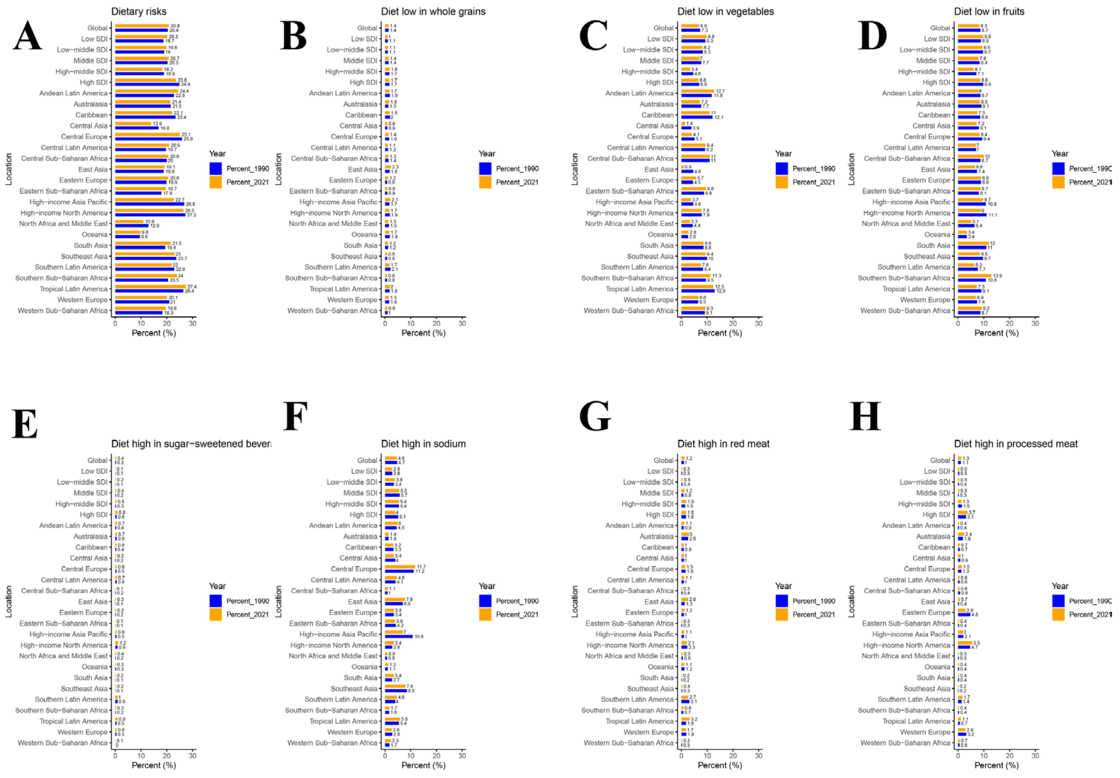

Supplementary Figure S3. Proportion of CKD-related Deaths attributable to specific dietary components in 1990 and 2021, in global, 5 SDI and 21 GBD regions. (A) Dietary risks (B) Diet low in whole grains (C) Diet low in vegetables (D) Diet low in fruits (E) Diet high in sugar-sweetened beverages (F) Diet high in sodium (G) Diet high in red meat (H) Diet high in processed meat. CKD, Chronic kidney disease; SDI, Socio-demographic Index; GBD, Global Burden Disease.

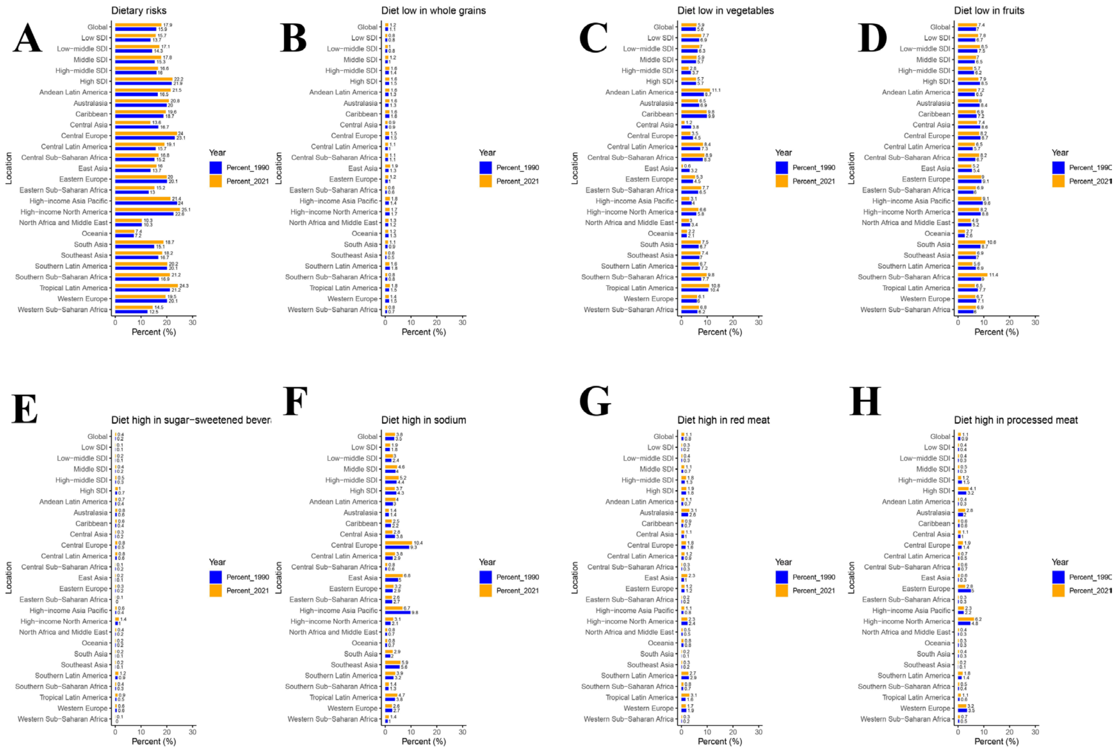

Supplementary Figure S4. Proportion of CKD-related DALYs attributable to specific dietary components in 1990 and 2021, in global, 5 SDI and 21 GBD regions. (A) Dietary risks (B) Diet low in whole grains (C) Diet low in vegetables (D) Diet low in fruits (E) Diet high in sugar-sweetened beverages (F) Diet high in sodium (G) Diet high in red meat (H) Diet high in processed meat. CKD, Chronic kidney disease; DALYs, Disability-Adjusted Life Years; SDI, Socio-demographic Index; GBD, Global Burden Disease.
